# Residential Proximity to High-Voltage Power Lines and Risk of Childhood Hematological Malignancies

**DOI:** 10.2188/jea.14.118

**Published:** 2005-03-18

**Authors:** Tetsuya Mizoue, Yasuhiro Onoe, Hiroshi Moritake, Jun Okamura, Shigeru Sokejima, Hiroshi Nitta

**Affiliations:** 1 Department of Preventive Medicine, Faculty of Medical Sciences, Kyushu University.; 2 Department of Pediatrics, Miyazaki Prefectural Hospital.; 3 Department of Pediatrics, Miyazaki Medical College.; 4 Institute for Clinical Research, National Kyushu Cancer Center.; 5 Department of Epidemiology and Health Care Research, Graduate School of Medicine and Faculty of Medicine, Kyoto University.; 6 National Institute for Environmental Studies.

**Keywords:** child, electromagnetic fields, leukemia, lymphoma, risk factors

## Abstract

BACKGROUND: Epidemiologic studies of electromagnetic fields and childhood cancers have focused on home exposure. The authors investigated whether residence in districts near high-voltage power lines is associated with childhood hematological malignancies, using small area analysis.

METHODS: Among 50,000 children in a city in Japan, 14 cases aged younger than 15 years were diagnosed with these malignancies in the period from 1992 through 2001. A total of 294 districts constituting this city were classified according to their proximity to high-voltage power lines (either 66 kV or 220 kV). Mantel-Haenszel rate ratio is used to calculate incidence rate ratio and its 95% confidence interval (CI).

RESULTS: Compared to districts of which no area fell within 300 m of high-voltage power lines, districts in which at least 50% of the area fell within 300 m of high-voltage power lines demonstrated an increased risk (incidence rate ratio: 2.2; 95% CI: 0.5-9.0). The association was strengthened for homes in which patients had resided for the longest interval of their lives (incidence rate ratio: 3.4; 95% CI: 0.9-13.2). Point-in-time measurements showed no increase in magnetic field levels for patient homes in districts near the lines.

CONCLUSION: An increased, albeit nonsignificant, risk of childhood hematological malignancies associated with residential proximity to high-voltage power lines warrants further investigations.

The association of exposure to extremely low frequency electromagnetic fields (EMF) with childhood cancers has been intensively studied. Wertheimer and Leeper first investigated the association using wire coding around homes as a surrogate of EMF exposure,^1^ while subsequent studies have estimated or measured home magnetic fields.^2^^-^^8^ Pooled analyses have shown a two-fold increase in risk for childhood leukemia associated with home magnetic fields of 0.3 *μ*T or 0.4 *μ*T or higher.^9^^,^^10^ Studies to date have mainly focused on exposure at home, based on either wire coding or EMF measurements.

High-voltage power lines (HVPLs), a strong and stable source of EMFs in residential environment, have been repeatedly linked to cancer risks.^11^ Studies on this issue have used distance from home to HVPLs, or estimated magnetic fields based on distance, as an exposure index. However, accounting for the full range of activities of children outside home, including at school, in parks, at shops, or in between, suggests that children who reside in districts near HVPLs face greater risk of exposure to higher levels of EMF originating from HVPLs than those who reside far from such lines. The proximity of the residential district generally, not home, to HVPLs may serve as an alternative index for EMF exposure relevant to health risk. To explore this question, we investigated whether residence in district near an HVPL is associated with increased risk of childhood hematological malignancies, using small area population statistics. The present study was proposed by a pediatrician (one of the authors), who plotted on a map homes of children who were diagnosed as having hematological malignancies in his department and suspected a link of these malignancies to residential proximity to HVPLs.

## METHODS

### Study region

The study region is a city having an area of 287 km^2^ located in southwest Japan, in which approximately 50,000 children aged 14 years or younger reside. The city consists of 294 small districts. The mean area per district is 0.96 km^2^. Eighty percent of the population is concentrated into two dense regions having a total area of 91 km^2^; the mean area per district in these regions is 0.35 km^2^. Two types of HVPLs have been identified; 66 kilo-voltage lines run through the city, including residential areas, while 220 kilo-voltage lines run along part of the perimeter of the outer city. Information on voltage was obtained from the local electric company. The power frequency for these lines is 60 Hz.

### Case ascertainment

We focused on childhood hematological malignancies. Without a population-based cancer registry in the study area, we obtained demographic and clinical information from pediatricians of two major hospitals (one in the city and another in an adjacent municipality) that treat hematological malignancies. Because there were no other medical facilities providing such care in the vicinity, all the patients with these diseases in the city were assumed to have been admitted to one of the two hospitals. Our study network as well as resources did not allow us to collect information on other types of childhood neoplasm including brain tumors, and adult cancers. The upper age limit of 14 years (diagnosis before the birth day of 15 years old) was chosen because this definition has been frequently used in EMF-childhood leukemia studies.^9^^,^^10^ During the 10-year period from 1992 through 2001, 14 children were diagnosed pathologically with acute lymphocytic leukemia (n=11), acute myeloid leukemia (n=2), and non-Hodgkin’s lymphoma (n=1). We contacted the parents of 10 cases through their physicians and obtained information on home addresses at the time diagnoses were made and the homes in which patients had lived for the longest interval during the period from birth through diagnosis. We obtained written consent from the parents. For two deceased cases and two cases involving patients who had moved to other regions, physicians provided only the names of the district where the patients resided at the time of diagnosis, omitting detailed addresses.

### Mapping

We used a map of the city drawn to a scale of 1 to 25,000 (the Geographical Survey Institute [“Kokudo-Chiri-In” in Japanese]), in which HVPLs are indicated. A research assistant drew boundaries of small districts, with reference to a detailed map drawn to a scale of 1 to 1,500 (Zenrin Co., Ltd.). The research assistant also drew lines 300 m on either side of the HVPLs on the map. Then, 83 districts having any area within these two lines were categorized in a semi-quantitative manner, according to the proportion of the area within 300 m of the HVPLs: most of the area included (over 80%); half or more of the area at this distance or closer to a HVPL (50 to 80%); less than half of the area at this distance or closer (10 to 49%); a small fraction of the area at this distance or closer (less than 10%). Other criterion was also used to classify the districts, but the area within 100 m of the HVPLs assigned only 2,791 children aged less than 15 years in the highest exposure group, which may be too few for analysis. Using the map, we measured the shortest distance from the nearest HVPL to patients’ homes for which full addresses were provided. If the home in which a patient resided for the longest interval was within 100 m of the HVPL (n=3), point-in-time measurements of magnetic fields were made in front of the gate of the home, using an EMDEX-II (Enertech Co., Ltd.; 40-800 Hz).

### Statistical analysis

We merged the upper two categories of exposure into a broader category entitled “more than half of the area at this distance or closer,” and the third and fourth categories into a category of “less than half of the area at this distance or closer” for analysis. We used population estimates of five-year age groups at the district level in 1998 (Nippon Statistics Center Co., Ltd.), calculated from age distribution for each district in 1995 (census data) and the total population for each district in 1998 (population registry data). We assumed that, in calculating person-year, the number of population in each age- and exposure-category was stable during the study period.

Mantel-Haenszel rate ratio was used to estimate age-adjusted incidence rate ratio (IRR) and corresponding 95% confidence interval (CI) for these exposure categories, compared to “no area at this distance or closer” group.

IRR = ∑ (A_1i_T_0i_/T_+i_)/ ∑ (A_0i_T_1i_/T_+i_),^12^

where A_1i_ is the number of exposed cases out of T_1i_ person-time units; A_0i_ is the number of unexposed cases out of T_0i_ person-time units; T_+i_ equals to T_0i_ plus T_1i_. A variance estimator for the logarithm (ln) of the Mantel-Haenszel rate ratio is

Var[ln(IRR)] = ∑ M_1i_T_1i_T_0i_/T_+i_^2^/(∑ A_1i_T_0i_/T_+i_)(∑ A_0i_T_1i_/T_+i_),

where M_1i_(=A_1i_+A_0i_) is the total number of cases in stratum i.^13^ These estimates were used to yield 95 percent confidence limits of exponential of In(IRR)±1.96 Var[In(IRR)]^1/2^.

We calculated the risk estimate both for homes at the time of diagnosis and for homes in which patients had resided for the longest intervals during their lives. In the latter analysis, we used the home address at the time of diagnosis for four patients whose residential information prior to diagnosis was lacking, and excluded two patients who had resided the longest intervals outside the study region.

## RESULTS

Table 1 is the list of study cases in relation to residential proximity to HPVLs. Table 2 shows the association between residential proximity to the HVPLs and the risk of childhood hematological malignancies for homes at the time of diagnosis and for homes where cases had resided for the longest intervals during their lives. Districts at least half of whose area fell within 300 m from HVPLs (accounting for 13% of all the children in the city) were associated with a two-fold increased risk for these malignancies, compared to districts with no area falling within 300 m of a HVPL (incidence rate: 4.5 versus 2.1 per 100,000 person-years; age-adjusted IRR=2.2; 95% CI: 0.5-9.0). The association was strengthened and approached statistical significance for homes in which patients had resided for the longest intervals during their lives (IRR: 3.4; 95% CI: 0.9-13.2).

**Table 1.** List of study cases in relation to residential proximity to high-voltage power lines in a city in Japan, 1992-2001.

Age at diagnosis (year)	Sex	Type	Proportion of the area within 300 m of HVPLs for the district where the cases resided		Status as of June 2002

			Home at diagnosis	Home where case resided longest period of time	
5	F	ALL	<50 %	other prefecture*	
3	M	ALL	0 %	0 %	
14	M	ALL	<50 %	>50 %*	
13	F	ALL	>50 %	>50 %	
0	M	ALL	0 %	0 %	
12	F	ALL	>50 %	>50 %	
5	F	NHL	<50 %	unknown	died
13	F	ALL	0 %	0 %*	
2	F	ALL	<50 %	<50 %	
3	F	ALL	0 %	other prefecture*	
13	F	ALL	0 %	unknown	moved
11	M	ALL	0 %	0 %	
12	M	AML	>50 %	unknown	moved
8	M	AML	<50 %	unknown	died

* : Different from the home address at diagnosis.

HVPL : high-voltage power line

ALL : acute lymphocytic leukemia

AML : acute myeloid leukemia

NHL : non-Hodgkin’s lymphoma

**Table 2.** Residential proximity to high-voltage power lines and childhood hematological malignancies in a city in Japan, 1992-2001.

Percent area*	No. of districts	Childhood population (1998)	No. of patients	Incidence rate per 100,000 p-y	Age-adjusted incidence rate ratio^†^	95% CI
Home at diagnosis						
>50%	39	6636	3	4.5	2.2	0.5 , 9.0
<50%	44	15586	5^‡^	3.2	1.6	0.5 , 5.1
No^§^	211	29087	6	2.1	1.0	

Home where case had resided for the longest intervals^||^						
>50%	39	6636	4	6.0	3.4	0.9 , 13.2
<50%	44	15586	3^‡^	1.9	1.1	0.3 , 4.7
No^§^	211	29087	5	1.7	1.0	

* : Proportion of area within 300 m of high-voltage power lines (66kv, 220kv)

† : Mantel-Haenszel rate ratio

‡ : Including one patient with non-Hodgkin’s lymphoma

§ : Reference category

|| : Excluding two patients who had resided for the longest intervals in other prefectures; classifying four patients who died or moved to other prefectures according to the home address at diagnosis

p-y : person-year

CI : confidence interval

Of four patients who had resided in districts in the highest exposure category, detailed addresses were provided for three. The distances from these three homes to the nearest HVPL were 76, 80, and 93 m; point-in-time measurements made at the gates of the respective homes were all 0.03 *μ*T.

Magnetic field levels were measured around two schools within 50 m of HVPLs in the school districts where school-age cases resided. Point-in-time measurements of magnetic fields showed readings of 0.35 *μ*T at one school gate and similar values on the same street, along which a HVPL passes overhead; and 0.16 *μ*T at another school gate, 34 m from a HVPL, and 0.32 *μ*T on the sidewalk opposite the gate.

## DISCUSSION

Applying small area analysis, we found an increased risk of childhood hematological malignancies among children who lived in districts near HVPLs. This suggests that the exposure index at the district level may serve as a useful predictor of the risk of childhood hematological malignancies.

The present study is unique in that EMF exposure was determined at the small district level. We assumed that children residing in districts near HVPLs were frequently exposed to higher levels of EMF originating from the HVPLs during daily activities, regardless of the location of their homes. One potentially important place where children are exposed to EMF is school. Even if homes are far from HVPLs, children may have notable EMF exposure at school if their school is situated near a HVPL. Although we did not collect information about school to which cases attended, our spot-measurement results suggest that some cases had higher EMF exposure at school than at home (since Japan has a school district system in which children living in a certain area generally attend a corresponding local school). A finding reported by Vistnes et al.^14^ supports our assumption: for children attending schools very close to a HVPL, the magnetic field exposure sustained during time spent at school contributed significantly to overall exposure. Magnetic fields may also be stronger at other places, including kindergartens, parks, shops, and areas in between.

There are methodological limitations in the present study. First, exposure categorization depends on the size of a district; districts of a larger area tend to fall into the category of districts having less than half of their area within a certain distance of HVPLs. Comparisons of highest versus low exposure groups may be less subject to this problem. Second, we did not consider power line characteristics, including line height and current levels, which determine the levels of EMF around HVPLs. Third, we did not adjust for potential confounding factors such as socioeconomic status, air pollution, and population mixing. Because none of these is an identified risk factor for leukemia, we believe that the present findings cannot be explained by confounding bias. Fourth, this study did not account for exposure from distribution lines or in-home wiring. However, exposure from these sources is ubiquitous and should distort an estimate only to null.

The proximity of a district to HVPLs correlates with the distance from a home in the district to HVPLs. Thus, we may compare the present results to those from studies using distance from homes to a HVPL^6^^,^^7^^,^^15^^-^^18^ or magnetic fields calculated based on the distance.^4^^-^^7^^,^^19^ Of these, two studies showed a clear association with reasonable statistical confidence. In Sweden, Feychting and Ahlbom^6^ reported an increased risk of childhood leukemia associated with living within 50 m of HVPLs (odds ratio: 2.9; 95% CI: 1.0-7.3). In Taiwan, Li et al.^18^ found an increased risk of leukemia among children who resided within 100 m of HVPLs (standardized incidence ratio: 2.43; 95% CI: 0.98-5.01). The risk estimate in the present study is compatible with these findings. Some other studies have reported limited findings that suggest a link between childhood leukemia or lymphoma and residential proximity to HVPL,^4^^,^^7^^,^^15^^,^^16^ while others have found no such association.^5^^,^^17^^,^^19^ Interestingly, a large-scale case-control study in the United Kingdom^19^ found an increased risk of childhood leukemia when an exposure group was defined as those residing in areas of broader proximity to HVPLs (within 400 m; odds ratio: 1.4).

In the present study, all measured magnetic fields were low (0.03 *μ*T) at the gates of homes of patients residing in a district near a HVPL. This result agrees with the finding of the study by Feychting and Ahlbom,^6^ who reported a relation between leukemia and distance or estimates of magnetic fields based on distance, but not with measured magnetic fields. Similarly, childhood leukemia has been linked to wire code but not to spot measurements.^2^^,^^3^ Such discrepancies have been ascribed to differences in the extent of agreement with past exposure; distance is a more relevant index of past exposure than measurements of magnetic fields.^20^ We offer another speculative hypothesis: that the distance from home to HVPL is a surrogate of exposure not only at home, but in the neighborhood environment, while EMF measurements at home reflect domestic exposure only.

Studies using EMF monitoring devices may be able to precisely evaluate exposure during daily activities. A study found a dose-response relation between monitored magnetic fields and childhood leukemia,^21^ with odds ratios beginning to increase from 0.03 *μ*T, while another study did not.^22^ The limitations of this type of study include a selection bias associated with study participation, changes in behavior after diagnosis, and inability to differentiate the nature of the EMF measured: whole-body exposure from surface or partial exposure.

The present study based on an exposure index at the district level provides additional evidence of the association between residential proximity to HVPL and childhood leukemia. This suggests that future studies should incorporate exposure assessments outside the home environment. Because the sample size was small and the present study area was chosen according to the proposal by a pediatrician, who suspected a linkage between childhood hematological malignancies and residential proximity to HVPL, confirmatory studies are needed which cover wider areas and include larger number of cases.
